# Modelling cancer outcomes of bone metastatic patients: combining survival data with N-Telopeptide of type I collagen (NTX) dynamics through joint models

**DOI:** 10.1186/s12911-018-0728-1

**Published:** 2019-01-17

**Authors:** Hugo Loureiro, Eunice Carrasquinha, Irina Alho, Arlindo R. Ferreira, Luís Costa, Alexandra M. Carvalho, Susana Vinga

**Affiliations:** 1 INESC-ID, Instituto Superior Técnico, Universidade de Lisboa, Rua Alves Redol, 9, Lisboa, 1000-029 Portugal; 2 IDMEC, Instituto Superior Técnico, Universidade de Lisboa, Av. Rovisco Pais 1, Lisboa, 1049-001 Portugal; 3 Instituto de Telecomunicações, Av. Rovisco Pais 1, Lisboa, 1049-001 Portugal; 4 Instituto Superior Técnico, Universidade de Lisboa, Av. Rovisco Pais 1, Lisboa, 1049-001 Portugal; 5 Instituto de Medicina Molecular, Av. Professor Egas Moniz, Lisboa, 1649-028 Portugal

**Keywords:** Joint models, Cancer studies, Fuzzy clustering, Bone metastasis, Survival analysis, Longitudinal analysis

## Abstract

**Background:**

Joint models (JM) have emerged as a promising statistical framework to concurrently analyse survival data and multiple longitudinal responses. This is particularly relevant in clinical studies where the goal is to estimate the association between time-to-event data and the biomarkers evolution. In the context of oncological data, JM can indeed provide interesting prognostic markers for the event under study and thus support clinical decisions and treatment choices. However, several problems arise when dealing with this type of data, such as the high-dimensionality of the covariates space, the lack of knowledge about the function structure of the time series and the presence of missing data, facts that may hamper the accurate estimation of the JM.

**Methods:**

We propose to apply JM for the analysis of bone metastatic patients and infer the association of their survival with several covariates, in particular the N-Telopeptide of Type I Collagen (NTX) dynamics. This biomarker has been identified as a relevant prognostic factor in patients with metastatic cancer, but only using static information in some specific time points.

**Results:**

We extended this analysis using the full NTX time series for a larger cohort of patients with bone metastasis, and compared the results obtained by the JM and the extended Cox regression model. Imputation based on fuzzy clustering was used to deal with missing values and several functions for NTX evolution were compared, such as rational, exponential and cubic splines.

**Conclusions:**

The JM obtained confirm the association between NTX values and patients’ response, attesting the importance of this time series, and additionally provide a deep understanding of the key survival covariates.

## Background

In medical research, longitudinal studies are often conducted to investigate disease evolution, to assess the effect of certain interventions (e.g. drugs or surgery), or to explore the association between certain risk factors and a clinical outcome. In these studies, patients are followed-up during a given period, and data are systematically collected. The obtained measurements can be static (time invariant), e.g. patient’s gender, but also time dependent, such as biomarkers evolution related with a given disease progression.

In these follow-up studies it is also relevant to analyse the time until an event of interest occurs, such as death or disease relapse, and investigate the association between patient’s characteristic and the outcome. In this context, survival analysis provides a statistical framework to analyse this type of data, through e.g. the estimation of Kaplan-Meier curves [1] and Cox proportional hazards regression models [2].

Although powerful to investigate static features, Cox models do not explicitly take into account the dependency of time series or repeated measurements data on the regression. In order to take into consideration time-variant features, an extended version of the Cox model can be used instead [3, Chapter 6]. Yet, this extension assumes that the time-dependent features are known for all time points and are measured without error. Using features that do not fulfil both of these requirements usually leads to bias on the results [4].

As a response to these limitations, joint models (JM) for longitudinal and survival data [5] are becoming increasingly popular in biostatistical literature to analyse clinical data with both time-variant and invariant features. The framework of this approach is to model the time-variant data with a linear mixed-effect model [6], whereas survival and time-invariant features are modelled with a Cox regression [2]. Examples of the application of JM can be found for various types of clinical data. They have been applied to HIV/AIDS [7], leukaemia [8], prostate cancer [9], breast cancer [10, 11] and lung cancer [12], to name a few.

Although adequate to estimate the association between the covariates and the times to the event of interest, the analysis with JM may become challenging due to mainly two factors: 1) the high variability of patients’ trajectories; and 2) missing information in the time-varying features. In an attempt to correctly model the high variability of patient’s data, cubic splines, that allow high flexibility in the longitudinal model, can be used [13]. Regarding the problem of missing values, many techniques have been proposed, namely, removing patients with missing values or extrapolating the missing values using previous information [14, 15], performing multiple imputation [16] or using fuzzy clustering-based techniques [17].

The aim of the present study is to extend existing models for bone metastatic patients disease progression using JM, by taking into account biomarkers time series.

Bone metastases are a common finding in patients with metastatic cancer, affecting up to 70% [18] and 90% [19] of patients with advanced breast and prostate cancers, respectively. Bone metastases are clinically relevant because they increase patients’ morbidity, manifested as bone pain, bone fractures or other bone complications, collectively referred to as skeletal related events [20]. The metastatic spread and subsequent establishment of cancer cells in the bone occurs after a complex interplay between cancer cells and the bone microenvironment [21]. In this process, cancer cells reorchestrate the fine-tuned equilibrium between bone-forming (osteoblasts) and bone-degrading (osteoclasts) cells to activate the bone metabolism and benefit from growth factors previously entrapped in the bone matrix - this process leads to a positive feedback loop of further tumour growth and added bone metabolism activation. Bone metabolism, either physiologic or pathologic (e.g., on the course of bone metastases), releases several by-products, namely from collagen breakdown, that are amenable of quantification in serum and urine. These by-products are collectively referred to as bone remodelling markers, and one of the most studied of these fragments is the N-telopeptide of type I collagen (NTX) [22]. The quantification of NTX and other bone remodelling markers allows to monitor overall bone metabolism, and thus to capture the overall disruptive effect of metastases in bone [23, 24].

In this context, we analyse a clinical dataset of bone metastatic cancer patients fully described in [24] but now taking into account the overall NTX evolution and not only specific time points (e.g. 3 or 12 months after the beginning of therapy). The application of JM coupled with fuzzy clustering-based imputation methods illustrates the advantages of using the full time series and supports the hypothesis of NTX clinical use as a biomarker for the disease.

## Methods

To understand the relationship between NTX and the death of bone metastatic cancer patients, a combined analysis of longitudinal and time-to-event data is performed. For the survival analysis the widely known Cox’s regression model [2] is briefly described. The linear-mixed effects (LME) models, [6], are introduced as one of the techniques used to model longitudinal data. Finally, the JM [5] and extended Cox [3, Chapter 6] regression, which combines survival and longitudinal data, are defined.

### Survival analysis

Survival analysis is a statistical technique used to study the time until an event of interest occurs. The event can be death, the relapse of a disease or the failure of some electronic component.

An important feature of survival analysis is that the event of interest may not be observed in all the patients under study. For example, if the event of interest is death, some patients can be still alive at the end of the study so we do not known the exact event time. Such survival times are named *censored*, to express that the study ended before the event of interest occurred.

In order to formalise survival models, we start by introducing some notation. Let *n* be the number of patients and $T^{*}_{i}$ a random variable representing the true event time for the *i*^*th*^ patient, with *i*=1,…,*n*. The observed event times are given by $T_{i}=\min (T^{*}_{i},C_{i})$, where *C*_*i*_ is the censoring time of patient *i*. The survival function *S*_*i*_(*t*) represents the probability of patient *i* surviving beyond time *t*, that is, *S*_*i*_(*t*)=*P*(*T*_*i*_>*t*), with *t*≥0. The probability that the event is experienced by the *i*^*th*^ patient, within a small time interval [*t*,*t*+*dt*), knowing that he has survived up to time *t*, is given by the hazard function *h*_*i*_(*t*):

1 $$ h_{i}(t) = {\lim}_{dt\to0^{+}}\frac{P\left(t\leq T_{i} < t + dt | T_{i} \geq t\right)}{dt}.  $$

A very popular statistical method used in survival analysis is the *Cox regression model* [2], which assumes that the effect that each feature has on the patient’s survival function is constant over time and postulates the hazard function as

2 $$ \begin{aligned} h(t; \boldsymbol{w}) = h_{0}(t)\exp\left\{\boldsymbol{\gamma}^{T}\boldsymbol{w}\right\},  \end{aligned}  $$

where *h*_0_(*t*) is the baseline hazard function, ***w***=(*w*_1_,*w*_2_,…,*w*_*p*_)^*T*^ is the patient’s time-invariant feature vector and ***γ*** are unknown regression coefficients.

### Longitudinal data analysis

Longitudinal data, comprising repeated measurements of patients over time, arise frequently in clinical studies. The main goal of a longitudinal study is the characterisation of temporal changes of some response of interest, for example to uncover the predictors of a given medical condition.

An important class of models that can take into account the variability among individuals and the average trends of the populations is the *linear mixed-effects models* (LME) [6]. These models comprise two components, a population-specific component, denoted as fixed effects, and a patient/group-specific component, describing the patient’s deviation from the population mean, termed as random effects.

The LME model for the *i*^*th*^ patient is given by

3 $$ y_{i}(t) = \boldsymbol{x}_{i}^{T}(t) \boldsymbol{\beta} + \boldsymbol{z}_{i}^{T}(t) \boldsymbol{b}_{i} + \epsilon_{i}(t),   $$

where *y*_*i*_(*t*) is the observed feature at time *t* for the *i*^*th*^ patient, ***x***_*i*_(*t*) is the fixed effects design matrix, ***β*** is the fixed effects vector, ***z***_*i*_(*t*) is the random effects design matrix, ***b***_*i*_ is the random effects vector, and *ε*_*i*_(*t*) is the random observation error. The model assumptions are the following: ***b***_*i*_∼*N*(0,***D***) and *ε*_*i*_(*t*)∼*N*(0,*σ*^2^), where ***b***_*i*_ and *ε*_*i*_(*t*) are independent between groups and between each other, ***D*** is the random effects covariance matrix, and *σ*^2^ is the variance of the error.

In longitudinal analysis, the shape of the patients’ trajectories can be highly non-linear, severely hampering the accurate estimation using simpler models. One possible solution is to adopt more complex functions in the LME models, such as cubic regression splines [13], which provide good estimators for the mixed and random effects:

4 $$ {\begin{aligned} NC\left(t,k,\boldsymbol{\beta}, \boldsymbol{b}_{i}\right) = \left(\beta_{j} + b_{ij}\right) {NC}_{j}(t), \textrm{ with}\ s_{j} < t \leq s_{j+1}, ~ j = 0,\dots,k, \\ \end{aligned}}  $$

where *s*_0_=*t*_0_ and *s*_*k*+1_=*t*_*f*_, *NC*_*i*_(*t*)=*a*_1*i*_*t*^3^+*a*_2*i*_*t*^2^+*a*_3*i*_*t*+*a*_4*i*_ is the natural cubic spline function for time point *t*, *k* amounts for the number of knots of the spline with locations *s*_1_,*s*_2_,…,*s*_*k*_, and, *t*_0_ and *t*_*f*_ are the initial and final points of the time series, respectively.

### Longitudinal and time-to-event analysis

The aforementioned Cox regression model [2] is used to investigate if the features of interest are associated with the event under study, assuming that the features do not change over time (are time-invariant). While this restriction can be adequate in some medical studies, the analysis of time-variant features might be of interest in other applications. In fact, there are several examples in the literature confirming these associations, e.g. CD4 counts and the development of AIDS [25].

*Joint models* (JM) for longitudinal and time-to-event data, or, simply joint models, were developed to analyse both time-invariant and time-variant features and their relationship with the event process [5]. JM combine a longitudinal model, to address time-variant features, with a survival model that takes into account the time until the event.

To formalise the model, let *m*_*i*_(*t*) denote the true and unobserved value of the time-variant feature for patient *i* at time *t*. To obtain the association of the features to the event, the survival sub-model is given by

5 $$ h_{i}\left(t\left|\mathcal{M}_{i}(t), \right.\boldsymbol{w}_{i}\right) = h_{0}(t) \exp\left\{\boldsymbol{\gamma}^{T} \boldsymbol{w}_{i} + \alpha m_{i}(t)\right\}, \textrm{ for}\ t > 0,   $$

where $\mathcal {M}_{i}(t) = \left \{m_{i}(s), 0 \leq s < t\right \}$ is the true unobserved time-variant feature until time *t* and *α* denotes the association of the time-variant feature to the event.

In the Cox models the baseline hazard is often left completely unspecified to avoid misspecification of the distribution of the survival times [5]. However, in JM the baseline hazard function, *h*_0_(*t*), must be specified to avoid the underestimation of the standard error values [5]. A standard option is to use a parametric distribution, such as the Weibull or the log-normal. Alternatively, one can also specify *h*_0_(*t*) in a more flexible way by using e.g. stepwise-constant functions or B-splines [5].

JM uses the true and unobserved time-invariant feature *m*_*i*_(*t*), while in most cases only specific measurements *y*_*i*_(*t*) are known. The relationship between *m*_*i*_(*t*) and the observed values *y*_*i*_(*t*) is given by the longitudinal sub-model, expressed as

6 $$ \begin{cases} \begin{aligned} y_{i}(t) & = m_{i}(t) + \epsilon_{i}(t) \\ m_{i}(t) & = \boldsymbol{x}_{i}^{T}(t)\boldsymbol{\beta} + \boldsymbol{z}_{i}^{T}(t)\boldsymbol{b}_{i} \\ \end{aligned}, \end{cases}   $$

with $\boldsymbol {b}_{i} \sim \mathcal {N}(0,\boldsymbol {D})$ and $\epsilon _{i}(t) \sim \mathcal {N}\left (0,\sigma ^{2}\right)$.

The estimation of JM usually involves Expectation-Maximization (EM) and Quasi-Newton algorithms to minimise the log-likelihood [7] or Bayesian approaches such as Markov Chain Monte Carlo (MCMC) methods [8]. Although JM allows the integration of time-variant and survival data, their inference is more computationally intensive, which can be a disadvantage in studies with a large number of patients with complex time-variant features [5].

#### Extended Cox model

The *extended Cox model* [3, Chapter 6] is an extension of Cox regression by introducing time-variant features directly in the hazard function. In this model, the time-variant features are considered as step-like functions, with jumps at each of the measurement times [3, Chapter 4]. Under this assumption, for any given patient with observations at time points *t*_1_,…,*t*_*N*_, the value of the time-variant feature at time *t*_*y*_, with *t*_*r*_<*t*_*y*_<*t*_*r*_+1, is given by the last registered measurement *t*_*r*_. The hazard function of the extended Cox model is given by

7 $$ h_{i}(t; \boldsymbol{w}) = h_{0}(t)\exp\left\{\boldsymbol{\gamma}^{T}\boldsymbol{w}(t)\right\},   $$

where ***w***(*t*) denotes a vector of both time-variant and time-invariant patient features. This hazard function is very similar to Eq. (2) but with ***w***(*t*) changing over time.

Even though the extended Cox model can handle time-variant features, it is not appropriate to deal with patient biomarkers. This is due to the fact that it assumes that the time-variant features are predictable processes, measured without error and that their full path is completely known [5].

The main difference between the extended Cox model and the JM is that in the latter the time-variant features are described by a LME model. More specifically, the extended Cox model considers that the time-dependent variables ***w***(*t*) are step-like functions with jumps at each of the measurements. This approach is far from ideal in our application since the biomarkers in study are continuous functions that change over time and not only in certain time-points. This less than ideal modelling of the time-variants may lead the derived results to be substantially biased [4]. The JM take a different approach by modelling the time dependent variable with a LME model, therefore creating a model without the aforementioned assumptions regarding the shape of the time-variant features [5].

### Missing data imputation

Missing data is a problem that affects almost all clinical studies [14, 16]. Several methods were developed to cope with this challenging issue, which includes the following strategies explored more deeply in the present study: (i) omitting or ignoring the corresponding missing entries; (ii) imputation based on the Last Observation Carried Forward (LOCF); (iii) imputation based on the Optimal Completion Strategy (OCS) [26] using Fuzzy Short Time Series Clustering (FSTS) [27].

The first is the simplest strategy and corresponds to omitting or ignoring the missing values, which may lead to a different sampling scheme for each patient of the cohort but does not constitute a problem for the inference of LME models [6].

The second approach is based on imputing the missing value with the last known observation of the same patient, i.e., the Last Observation is Carried Forward (LOCF) [15, Chapter 13].

Finally, the third method is based on time series clustering. The rationale of this approach is that if the patients are previously clustered based on their time-varying characteristics, one can use the group information to impute missing data. More specifically, if we assume that the patients in a given cluster are similar under a specific metric, it is reasonable to impute missing values based on patients that are ‘close’ and for whom complete information is available.

Although clustering algorithms abound in the literature, methods for short time series data are still scarce. We will focus on Fuzzy Short Time Series Clustering (FSTS) [27] given its previous successful application in the context of survival data [17]. The FSTS algorithm treats the time series as piecewise linear functions and uses the slope in each of the segments as input for the distance function.

We can then combine FSTS with and Optimal Completion Strategy (OCS) to perform the imputation of the missing values, a procedure fully described in [17, 28].

**Fig. 1:**
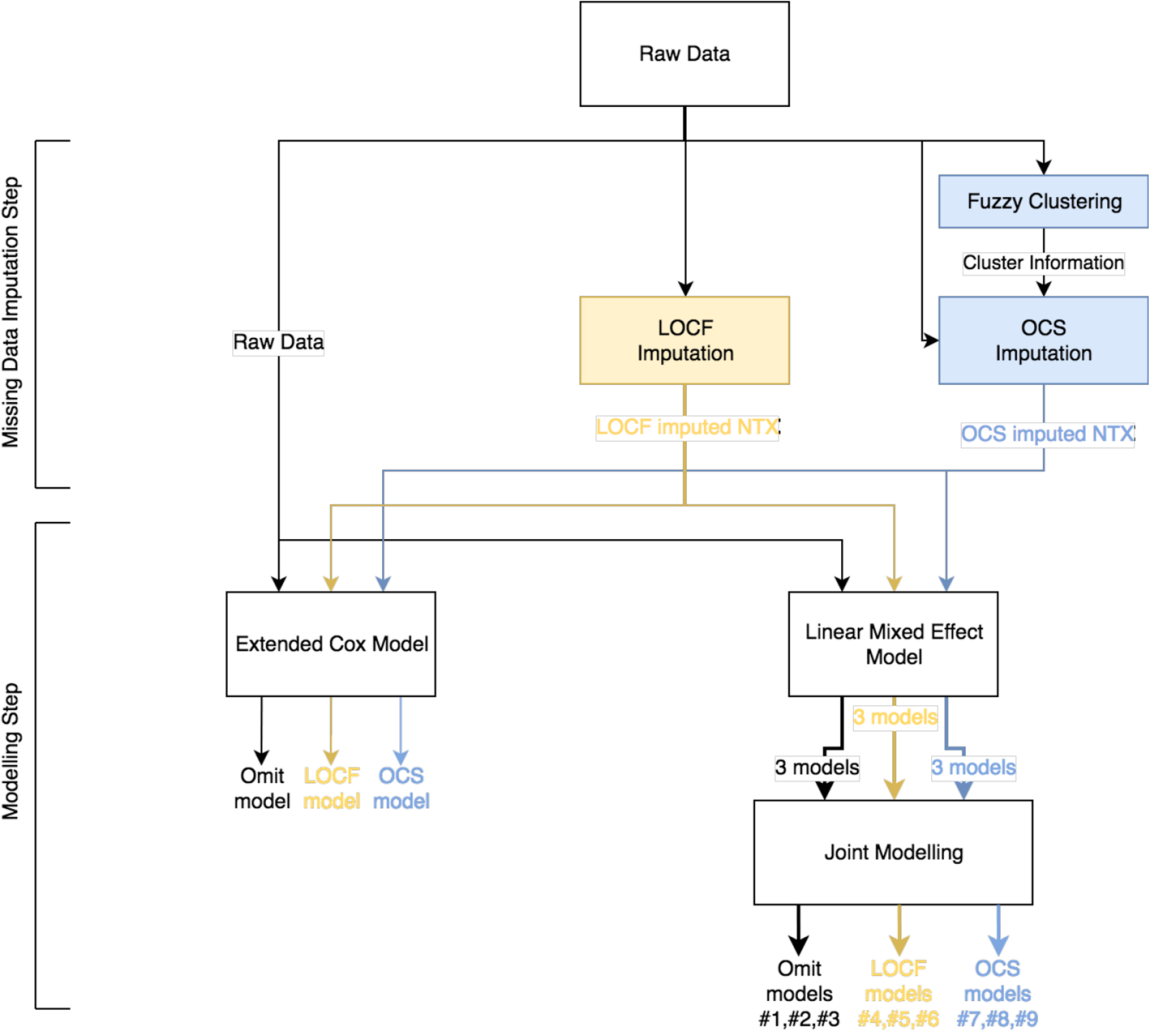
Global approach flowchart. Representation of the analytical approach followed. The raw data (with missing values) is processed in parallel by three different imputation techniques: omitting or ignoring the corresponding missing entries (Omit), Last Observation Carried Forward (LOCF) and Optimal Completion Strategy (OCS). These three imputed datasets are then separately analysed by the Extended Cox Model and the Joint Models (JM), thus generating three Extended Cox models and nine JM

In order to improve the methodology used, a flowchart (Fig. 1) is presented.

### Bone metastatic data

The dataset used in this work is based on a longitudinal cohort study carried out in Santa Maria Hospital (Lisboa, Portugal). In this dataset, patients with several types of primary cancers and bone metastases were followed while receiving primary cancer treatment plus a bone targeted agent – bisphosphonates (BPs).

The cohort includes 147 patients and the data comprises survival status, several time-invariant features, and a time series concerning N-telopeptide of type I collagen (NTX) measurements. The measurements of the NTX biomarker were carried out at the baseline, and at 1, 3, 6, 9 and 12 months (denoted by NTX0, NTX1,..., NTX12) after starting BPs. For details concerning how NTX was collected and determined see [24]. In Table 1 a detailed description of the number of patients per type of cancer is presented.

**Table 1:** Number of patients per type of cancer that have NTX measurements on month *t* (NTX*t*)

Cancer type	Baseline	NTX1	NTX3	NTX6	NTX9	NTX12
Breast	90	67	58	48	32	26
Prostate	26	20	17	15	11	5
Others	31	18	15	9	5	3
Totals	147	87	90	72	48	34

Others include the following types of cancer: lung, kidney, gastric, sarcoma, hepatobiliary, bladder, endometrium, cervix, neuroendocrine, osteoblastoma and unknown primary tumor

Baseline time-invariant features included were: Age at Diagnosis, Sex, Type of Primary Cancer, X-Ray Pattern of Bone Lesions, Number of Skeletal Related Events (NSRE), Type of Skeletal Related Event (SRE), Estrogen Receptor, and if there are any metastasis outside the bone (denoted by ExtraMets). The time-to-event of interest is the survival time of the patients.

## Results

In this section several modelling strategies are applied to analyse longitudinal and survival data of the bone metastatic cohort described. A subset of this dataset containing 71 breast cancer patients was recently analysed considering time-independent NTX measurements [24]. In that study, it was considered that a value of NTX3 is elevated if it is larger than 100 nmol BCE/mmol creatinine and of NTX12 if it is larger than 64 nmol BCE/mmol creatinine.

In the present work, we extend this analysis to all types of cancer present in the cohort and we will include the whole NTX time series function, and not only isolated time points, in order to evaluate the predictive accuracy of this biomarker. The goal is to compare extended Cox regression with JM, identify subsets of features with prognosis significance and evaluate the impact of distinct imputation algorithms.

All the analysis were performed using the software R [29] and the associated libraries survival, nlme and JM [30–32]. Additional HTML files with the implemented code are available at http://web.ist.utl.pt/~susanavinga/JointModels/.

**Fig. 2:**
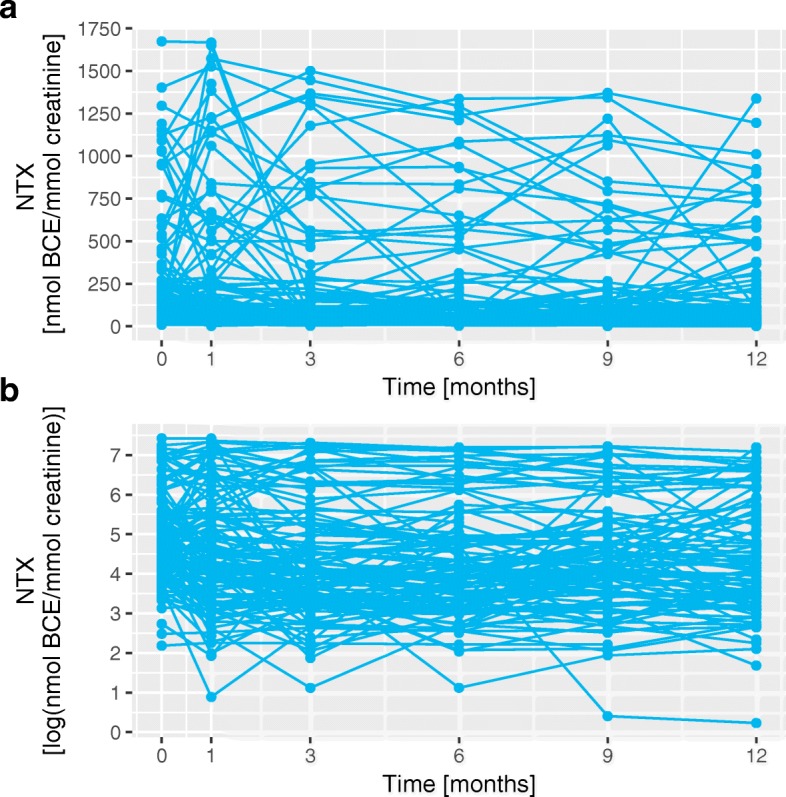
Comparison between NTX and log(*NTX*) trajectories. Graphical representation of the values of NTX and log(NTX) for all the patients. Panel **a** represents the original values and panel **b** represents the log-transformed values

Since we are now considering all types of primary cancers, features that are exclusive of a single type, like Estrogen Receptor, were not included in the analysis. The time-variant feature, NTX, is taken into account, and the real values of NTX3 and NTX12, as well as their log-transform, are analysed. The log-transform was used to reduce the disparity in NTX values between patients. This disparity is illustrated in Fig. 2 where the NTX values of each patient are plotted.

### Cox regression analysis

The first step in the analysis was to perform a univariate study similar to that of [24] where we used the Log Rank test [33] for categorical features and the Wald test to evaluate the statistical significance of the Cox regression coefficients [2].

The obtained *p*-values for each feature are presented in Table 2, where the age at diagnosis, the sex and extra metastasis are statistically significant variables in all the tests preformed.

**Table 2:** Log Rank and Wald tests *p*-values for each feature

	Feature	*p*-value	*p*-value
		Log rank	Wald test
Baseline	Age Diagnosis	-	**0.0028**
	Sex	**0.0140**	**0.0148**
	Primary Cancer	0.0872	-
	X-Ray Pattern	0.1264	-
	NSRE	0.8027	0.5627
	ExtraMets	**0.0161**	**0.0171**
Longitudinal	NTX3	-	0.4438
	log(NTX3)	-	**0.0220**
	NTX3 >100	0.1615	0.1515
	NTX12	-	0.0737
	log(NTX12)	-	0.0533
	NTX12 >64	**0.0171**	**0.0200**

The features were divided in Baseline (Time-independent) and Longitudinal (Time-dependent). The values in bold are statistically significant for a significance level of 5%

Regarding the time-variant features, log(NTX3) and NTX12 >64 obtained significant *p*-values for at least one of the tests performed, indicating that the actual measured NTX value should have prognosis value and not only the indication that it is high or low. Furthermore, NTX3 >100 appears to only have prognosis value for breast cancer patients since it did not obtain a significant *p*-value in the present analysis combining all the bone metastatic patients (contrary to the previous study [24]).

To perform the multivariate analysis, we then use the selected features from the univariate analysis to construct six multivariate Cox regression models, one for each NTX feature considered. The values of the regressor coefficients and their *p*-values for the multivariate models are represented in Table 3.

**Table 3:** Coefficents and *p*-values for the multivariate Cox regression model

Age diagnosis		Sex		ExtraMets			NTX	
Value	*p*-value	Value	*p*-value	Value	*p*-value	Type	Value	*p*-value
0.0220	**0.0088**	0.2485	0.3073	0.7823	**0.0017**	NTX3	0.0002	0.5875 ^a^
0.0209	**0.0127**	0.2421	0.3155	0.7393	**0.0034**	log(NTX3)	0.1211	0.2165 ^a^
0.0225	**0.0069**	0.2214	0.3614	0.7752	**0.0020**	NTX3 >100	0.1448	0.5831 ^a^
0.0184	0.1513	0.3709	0.3663	0.6223	0.0775	NTX12	0.0010	0.2225 ^b^
0.0177	0.1633	0.3647	0.3756	0.5563	0.1314	log(NTX12)	0.1638	0.2556 ^b^
0.0164	0.1910	0.5141	0.2312	0.4947	0.1806	NTX12 >64	0.6936	0.0781 ^b^

The NTX type column refers to the type of NTX feature used in each model fit. ^a^: Analysis using only patients with NTX3 measurement (106 patients with 86 events). ^b^: Analysis using only patients with NTX12 measurement (51 patients with 41 events)

In the first three models, the age at diagnosis and extra metastasis are significant, while sex and the NTX variables, obtained no significant *p*-values. The non-significance of the NTX values is not expected since NTX3 >100 was known to be significant in previous univariate and multivariate analysis [24]. This fact may be due to the difference in physiology between the cancer types, with NTX capturing bone degradation, which is more commonly observed in breast cancer patients.

### Joint models (JM) analysis

In further analyses, we consider the full NTX time series function in the models. To address the missing values problem, we apply the three methods described. The first two are straightforward and do not imply any specific preprocessing. The third requires two parameters: the number of clusters *c* and the partition coefficient *m*. The optimisation of these values for this dataset was already performed under OCS and FSTS [17] and we will use the same results, namely *c*=6 and *m*=1.3.

**Fig. 3:**
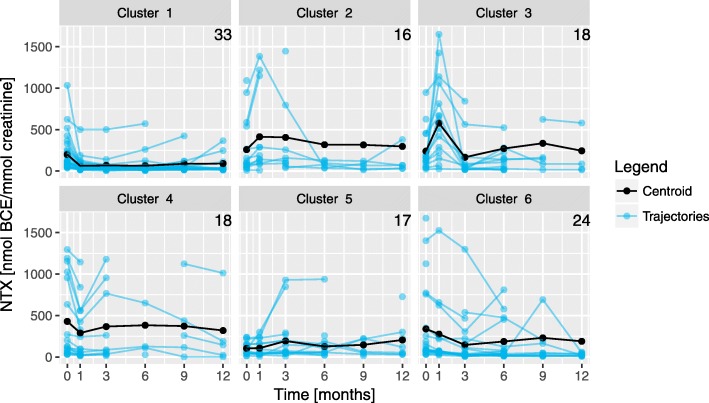
Centroids obtained in the FSTS clustering algorithm. Graphical representation of the six centroids obtained in the FSTS algorithm and NTX time series. The number in the top right corner of each plot is the number of patients with partition matrix coefficient of at least 0.75 for that cluster

The cluster centroids obtained are represented in Fig. 3, showing the high variability of NTX trajectories and heterogeneity between the clusters. For example, the clusters with the largest number of patients are 1 and 6 (33 and 24, respectively), which exhibit distinct NTX evolution. Cluster 1 represents patients where NTX0 values significantly decrease after the first month, remaining approximately constant afterwards. Cluster 6 represents patients whose NTX value decreases gradually over time from the baseline until 3 months, followed by an increase at 9 months. Based on these clusters, it is then possible to adopt the third imputation strategy.

**Fig. 4:**
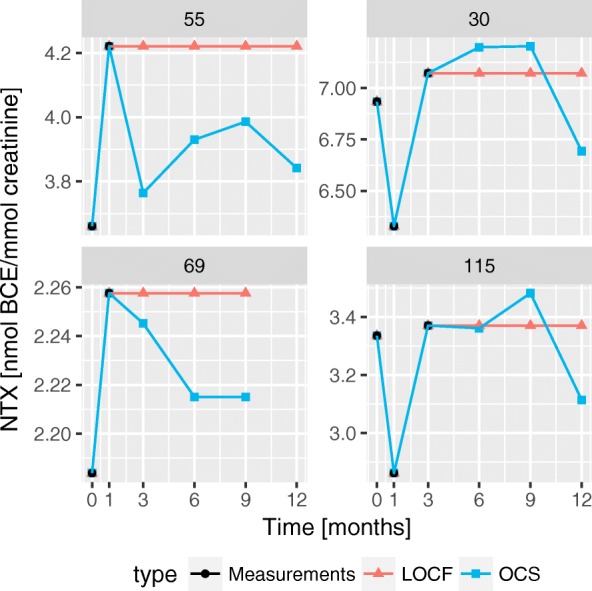
Comparison of the trajectories from different imputation techniques. Four patients were selected where the differences between the imputation techniques generate drastically different trajectories

These three strategies to treat the missing values may produce very different trajectories, as illustrated in Fig. 4. In this figure patients 30 and 115 have similar trajectories. The OCS algorithm imputes the missing values by increasing the NTX value between 3 and 9 months followed by a decrease at 12 months. While LOCF only extends the value at 3 months forward. For patients 55 and 69, their trajectories are also similar. With OCS generating trajectories with a maximum NTX value at 1 month that decays over time. While LOCF extends the maximum values onward, creating trajectories similar with step functions.

**Fig. 5:**
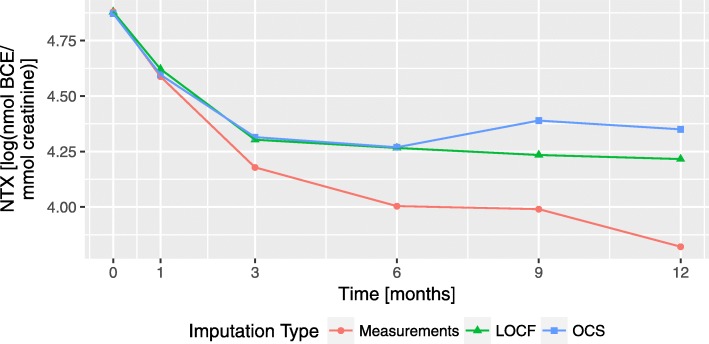
Mean value of NTX for each imputation type. The Measurements curve refers to the omission of the missing values, the LOCF curve to the values imputed with Last Observation Carried Forward and OCS to the Fuzzy Clustering approach using Optimal Completion Strategy

After the imputation of missing data, the next step is to correctly model NTX trajectories to be included in the LME model, i.e., determine a function that describes the longitudinal information given by this biomarker. To evaluate possible function types, we first represent the mean NTX trajectory for each of the imputation strategies, see Fig. 5. All three mean plots resemble a decaying function, similar to an exponential or a rational function. Since the OCS curve exhibits a slight increase (at 9 months) that cannot be modelled by neither of the aforementioned functions, spline models will also be included in the analysis.

Despite the mean NTX trajectory resembling a decaying function, we know that there is a high variability in the patient’s trajectories. To mitigate this and try to perform the best fit to all patients, we will specify ***z***_*i*_(*t*)=***x***_*i*_(*t*) in the LME model. This is, we will include in the models the same number of random effects as of fixed effects, thus increasing the number of degrees of freedom of the LME models and allowing for a better fit to each patient.

The expressions of the selected longitudinal models are represented in Table 4, where *δ* and *η* are used as tuning parameters. The optimal tuning parameters were obtained by performing separate Nonlinear LME (NLME) [6] fits.

**Table 4:** Longitudinal functions used in the JM

	#	Model	NTX_*i*_(*t*)
Omit	1	Rational	$ (\beta _{0}+b_{0i}) \,+\, (\beta _{1}+b_{1i})\frac {1}{(t+\delta)^{\eta }} + \epsilon _{i}(t), ~~~ \delta > 0, \eta > 0$
	2	Exponential	(*β*_0_+*b*_0*i*_) + (*β*_1_+*b*_1*i*_)exp(−*δt*)+*ε*_*i*_(*t*), *δ*>0
	3	Spline	(*β*_0_+*b*_0*i*_) + *NC* (*t*,2, (*β*_1_,*β*_2_,*β*_3_)^*T*^, (*b*_1*i*_,*b*_2*i*_,*b*_3*i*_)^*T*^) + *ε*_*i*_(*t*)
LOCF	4	Rational	$ (\beta _{0}+b_{0i}) \,+\, (\beta _{1}+b_{1i})\frac {1}{(t+\delta)^{\eta }} + \epsilon _{i}(t), ~~~ \delta > 0, \eta > 0$
	5	Exponential	(*β*_0_+*b*_0*i*_) + (*β*_1_+*b*_1*i*_)exp(−*δt*)+*ε*_*i*_(*t*), *δ*>0
	6	Spline	(*β*_0_+*b*_0*i*_) + *NC*(*t*,2, (*β*_1_,*β*_2_,*β*_3_)^*T*^, (*b*_1*i*_,*b*_2*i*_,*b*_3*i*_)^*T*^) + *ε*_*i*_(*t*)
OCS	7	Rational	$ (\beta _{0}+b_{0i}) \,+\, (\beta _{1}+b_{1i})\frac {1}{(t+\delta)^{\eta }} + \epsilon _{i}(t), ~~~ \delta > 0, \eta > 0$
	8	Exponential	(*β*_0_+*b*_0*i*_) + (*β*_1_+*b*_1*i*_)exp(−*δt*)+*ε*_*i*_(*t*), *δ*>0
	9	Spline	(*β*_0_ + *b*_0*i*_) + *NC*(*t*,2,(*β*_1_,*β*_2_,*β*_3_)^*T*^, (*b*_1*i*_,*b*_2*i*_,*b*_3*i*_)^*T*^) + *ε*_*i*_(*t*)

**Fig. 6:**
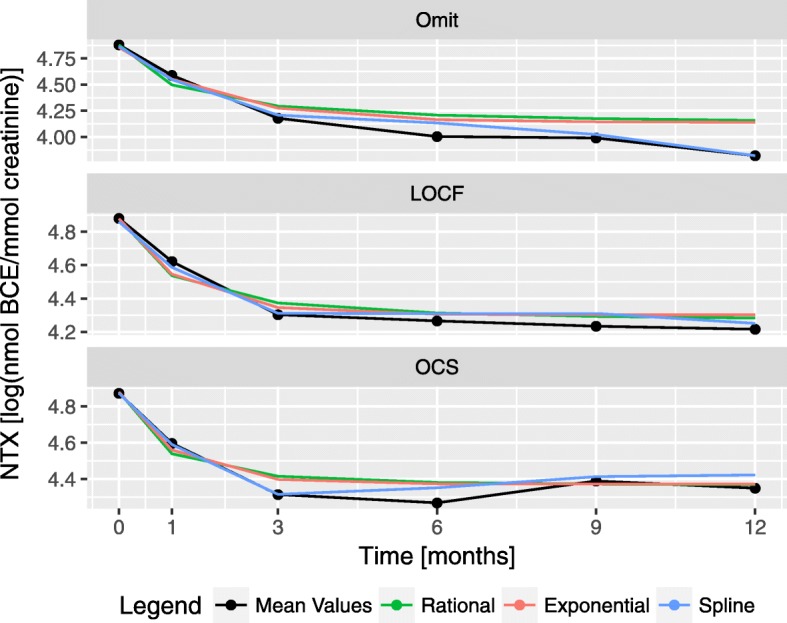
Population fits for the longitudinal models. The nine longitudinal fits from Table 4 are represented alongside the population mean for each of the imputation types

The mean NTX and the population fits of the LME models are presented in Fig. 6. All models correctly fit NTX mean trajectories, specially splines that are able to better capture non-linearities of the time series in the case where missing values are omitted.

Another requirement for the analysis using joint models is to define the baseline hazard function to avoid underestimation of standard error of parameter estimates [5]. In this work we use a piecewise-constant hazard function [5, Chapter 4].

Next, each of the longitudinal models obtained is used to generate nine JM, one for each longitudinal model and imputation type. The values and *p*-values for each of these JM are represented in Table 5. From the time-invariant features, the age at diagnosis and extra metastasis obtained significant *p*-values for all models. While sex was never significant. NTX variable was significant under models 3 and 6 to 9.

**Table 5:** Coefficients and *p*-values of the JM

	#	Age diagnosis		Sex		ExtraMets		log(NTX)	
		Value	*p*-value	Value	*p*-value	Value	*p*-value	Value	*p*-value
Omit	1	0.0185	**0.0152**	0.3160	0.1499	0.6655	**0.0035**	0.2093	0.0593
	2	0.0184	**0.0160**	0.3134	0.1530	0.6649	**0.0035**	0.2175	0.0638
	3	0.0184	**0.0147**	0.2655	0.2216	0.6051	**0.0085**	0.1095	**0.0442**
LOCF	4	0.0186	**0.0144**	0.3006	0.1672	0.6687	**0.0032**	0.1674	0.0755
	5	0.0186	**0.0147**	0.3005	0.1672	0.6673	**0.0032**	0.1692	0.0753
	6	0.0175	**0.0242**	0.2930	0.1820	0.6322	**0.0053**	0.1353	**0.0289**
OCS	7	0.0182	**0.0164**	0.3048	0.1612	0.6586	**0.0036**	0.1955	**0.0430**
	8	0.0181	**0.0166**	0.3046	0.1615	0.6575	**0.0037**	0.1962	**0.0430**
	9	0.0170	**0.0233**	0.3153	0.1496	0.6681	**0.0032**	0.1966	**0.0013**

In models 1 to 3 the missing values were omitted, in 4 to 6 LOCF was used to impute the missing values and in 7 to 9 OCS was used

The extended Cox model can also integrate longitudinal features into the hazard function but, contrary to the JM, does not require the longitudinal feature to be modelled using LME. In Table 6, the values and *p*-values of the coefficients for the extended Cox model are presented. The age at diagnosis and extra metastasis obtained significant *p*-values for both extended Cox models, while sex was not significant for any of the models. This is consistent with the previous results in both the multivariate Cox analysis and the analysis via joint models.

**Table 6:** Coefficients and *p*-values for extended Cox models

Model	Age diagnosis		Sex		ExtraMets		log(NTX)	
	Value	*p*-value	Value	*p*-value	Value	*p*-value	Value	*p*-value
Omit/LOCF	0.0171	**0.0256**	0.3049	0.1658	0.6485	**0.0047**	0.1401	0.0819
OCS	0.0172	**0.0232**	0.2963	0.1763	0.6458	**0.0048**	0.1629	**0.0496**

Since the extended Cox model considers the time-variant feature as a step function, with steps on each measurement, omitting missing values and LOCF generate the same extended Cox model

## Discussion

From a general point of view, models can aid medical professionals by unravelling which features are correlated with the event being studied, thus allowing for the identification of patients or groups of patients that are recognisable by their natural history or response to therapy. Such groups might impact the design of clinical trials and further feed translational research. At the same time, they can also be used to predict the response of new patients to the treatment, thus supporting medical research and the advancement of personalised treatments.

In order to address the importance of personalised medicine, in this work we analysed data from bone metastatic patients using different modelling strategies and missing data imputation techniques.

In the first two types of models, NTX was only analysed as a time-invariant feature. It obtained significant *p*-values for some univariate models, but when other features were included in the model, it lost its significance. The bone-cancer interaction, and ultimately the pattern of bone remodelling arising from this interaction, differs between tumours, both at the level of type of primary, but also at the specific biology of each tumour. In addition, each bone biomarker reflects different biochemical processes, with NTX better capturing the resorption part of bone remodelling and thus informing more accurately about tumours with predominately lytic or mixed lesions [22]. This is the case of breast cancer, but not, e.g., prostate cancer. Our findings using this data modelling approach support this view, that while NTX measurements might be informative in breast cancer, they do not inform about all cancer types.

Following the multivariate analysis, nine JM and two extended Cox models were analysed. In general the joint and extended Cox models obtained lower *p*-values for NTX than the multivariate Cox models. With all three of the JM with the spline modelling (models 3 and 6 to 9) obtaining a significant *p*-value for the value of log-transformed NTX. This result is different from the previous results, in that it shows that the instantaneous NTX value has prognostic value[24]. Indeed, even in predominantly bone forming lesions (as in prostate cancer), bone resorption is still present. Thus, this more accurate data modelling technique of NTX seems to be able to derive prognostic information from NTX variation in the complete spectrum of types of cancer.

The inclusion of the longitudinal modelling step in the joint model led to significant *p*-values for the NTX time series, which is consistent with the extended Cox regression model. Further analysis about the intrinsic knowledge that can be extracted from the estimated parameters will be addressed in the future.

When taking into account all types of cancers, only the joint models analysis was able to find a prognostic association between NTX and survival outcomes. Consequently, this finding suggests that the prognostic value of NTX is clearer as a time-varying feature, when compared to its modulation only as a time-invariant feature. Although providing a more accurate description, JM are more computational demanding and also require more parameters, which may make them prone to overfitting and should be taken into account when modeling clinical data.

## Conclusions

In this work we compared several modelling strategies that couple survival with longitudinal data. In particular, we analysed the relationship between NTX biomarker measurments and the survival times of bone metastastic patients. In previous analysis NTX was converted from a numerical feature into a categorical one whose values indicate only if NTX level was high or low [23, 24], in order to have clear cut-off criteria for direct application in the clinic. The time-variant nature of NTX was therefore ignored, with each measurement being treated as a different, independent feature. In this study we extend these models by considering the value of NTX a time-dependent feature when using survival analysis methods. A univariate analysis was first performed to evaluate the statistical significance of features. Based on the features selected, six multivariate Cox regression models were analysed, for each NTX. The results show that, for three of the models sex and NTX features were not statistically significant. To conduct the JM analysis the full NTX time series was considered. Different techniques to solve the problem of missing values were performed. Results exhibit that the instantaneous NTX value had prognostic value, contradicting the previous results obtained. Moreover, the results illustrate the advantages of Joint Models and their potential to identify relevant biomarkers with application in oncological studies.

## Abbreviations

BCE: Bone collagen equivalents
BPs: Bisphosphonates
EM: Expectation-maximization
FSTS: Fuzzy short time series clustering
JM: Joint models
LME: Linear-mixed effects
LOCF: Last observation carried forward
MCMC: Markov chain Monte Carlo
NTX: N-telopeptide of type I collagen
OCS: Optimal completion strategy
SRE: Skeletal related event
